# Prediction of Posterior Communicating Artery Aneurysm Rupture Risk: A Multivariate Analysis of Aneurysm and Surrounding Arterial Morphological Factors

**DOI:** 10.3390/jcm15103783

**Published:** 2026-05-14

**Authors:** Minu Nahm, Shin-Woong Ko, Hyeong-Joong Yi, Hyeong-Joon Chun, Min-Kyun Na, Young-Jun Lee, KyuNam Kim, Sang Hyung Lee, Jaiyoung Ryu, Simon Song, Kunhee Han, Kyu-Sun Choi

**Affiliations:** 1 Department of Neurosurgery, Hanyang University Medical Center, Hanyang University College of Medicine, Seoul 04763, Republic of Korea; nmw9009@yuhs.ac (M.N.); hjyi8499@hanyang.ac.kr (H.-J.Y.); tdy815@hanyang.ac.kr (H.-J.C.); naminkyun@hanyang.ac.kr (M.-K.N.); 2 Center for Precision Medicine Platform Based on Smart Hemo-Dynamic Index, Seoul 04763, Republic of Korea; pakky100@naver.com (S.-W.K.); nslee@snu.ac.kr (S.H.L.); jryu@korea.ac.kr (J.R.); simonsong@hanyang.ac.kr (S.S.); 3 Department of Neurosurgery, Hanyang University Guri Hospital, 153 Gyeongchun-ro, Guri 11923, Republic of Korea; 4 Department of Radiology, Hanyang University Medical Center, Hanyang University College of Medicine, Seoul 04763, Republic of Korea; yjleeee@hanyang.ac.kr; 5 Department of Anesthesiology and Pain Medicine, Hanyang University Medical Center, Hanyang University College of Medicine, Seoul 04763, Republic of Korea; knkim9@hanyang.ac.kr; 6 Department of Neurosurgery, Jeju National University Hospital, Jeju National University College of Medicine, Jeju 63241, Republic of Korea; 7 Department of Mechanical Engineering, Korea University, Seoul 02841, Republic of Korea; 8 Department of Mechanical Convergence Engineering, Hanyang University, Seoul 04763, Republic of Korea

**Keywords:** intracranial aneurysm, posterior communicating artery aneurysm, internal carotid artery, aneurysm rupture, surrounding artery

## Abstract

**Background/Objectives**: Recent studies have increasingly focused on the morphological characteristics of surrounding arteries as rupture predictors, particularly because these vessel configurations remain stable before and after aneurysm rupture, providing a reliable anatomical substrate for risk assessment. This study aimed to identify independent predictors of rupture by evaluating both aneurysmal and internal carotid artery (ICA) morphological characteristics. **Methods**: We retrospectively analyzed imaging data from 64 patients with posterior communicating artery (PcomA) aneurysms who underwent treatment at a single tertiary center between 2018 and 2022, including 25 ruptured aneurysms (39.1%). Only treated aneurysms were included to ensure the availability of high-quality pre-treatment digital subtraction angiography (DSA) suitable for three-dimensional (3D) reconstruction and centerline-based analysis. Seventeen aneurysm morphological parameters and thirteen ICA-related parameters were measured. Because time-to-event data were not available, logistic regression analysis was performed with rupture status as the outcome variable. Receiver operating characteristic (ROC) curve analyses were conducted to evaluate discriminative performance. **Results**: Multivariate logistic regression revealed that three ICA-associated factors—the tortuosity of the communicating ICA segment (Tcco), the ICA cross-sectional area at the PcomA origin (Pcs), and the angle between the ICA and PcomA (θ2)—were independently associated with rupture. Among aneurysm-related factors, Maximum 3D Diameter remained significantly related to rupture risk. ROC analyses demonstrated that Maximum 3D Diameter had the highest discriminative value (AUC 0.779; cut-off 7.805 mm), followed by Pcs, Tcco, and θ2. **Conclusions**: Both aneurysm morphology and the anatomical configuration of surrounding arteries significantly contribute to rupture risk in PcomA aneurysms. Incorporating parent-vessel morphological features into rupture-risk assessment may enhance patient-specific decision-making.

## 1. Introduction

Aneurysm rupture causes a subarachnoid hemorrhage (SAH), often resulting in poor outcomes or death [1]. Therefore, it is critical to decide whether to treat or monitor an unruptured aneurysm. Advancements in diagnostic techniques and the aging population have led to an increase in the detection of unruptured intracranial aneurysms [2]. Posterior communicating artery (PcomA) aneurysms represent 9–17% of cases, and their rupture risk is higher than that of aneurysms in other locations [3,4].

In previous studies, morphological parameters of aneurysms, such as size, aspect ratio (AR), size ratio (SR), and bleb formation, were significantly associated with the rupture of PcomA aneurysms [5,6]. However, some studies have highlighted significant changes in aneurysm morphology during rupture and pointed out the limitations of aneurysm morphology as a predictive marker of rupture [7,8].

In contrast, the morphological configuration of the surrounding arteries remains largely unchanged before and after rupture. This geometric stability, along with the important role of hemodynamic stress and vascular remodeling in the pathophysiology of aneurysm formation and rupture, has driven increasing interest in surrounding arterial morphology as a potential predictor of aneurysm rupture [9]. Previous studies have identified morphological parameters of surrounding arteries in PcomA aneurysms, including the angle between the internal carotid artery (ICA) and PcomA, the angle between the ophthalmic and communicating segments and the diameter of the ICA, as factors associated with aneurysm formation or rupture [4,10,11]. Building on this concept, this study aimed to predict the rupture risk of PcomA aneurysms by evaluating the morphological factors of both aneurysms and surrounding arteries.

## 2. Materials and Methods

### 2.1. Ethics Approval and Patient Consent

This retrospective study and all associated procedures were conducted in accordance with the ethical standards of the Institutional Research Committee and the principles outlined in the Declaration of Helsinki. The study protocol was reviewed and approved by the Institutional Review Board of Hanyang University Hospital (IRB approval number: HYUH2023-04-028). Given the retrospective nature of the study and the use of fully anonymized data, the requirement for informed consent from individual participants was waived by the IRB.

### 2.2. Study Population and Clinical Characteristics

We retrospectively reviewed the clinical and imaging data of patients with PcomA aneurysms who were treated between January 2018 and December 2022. The inclusion criteria were adult patients who underwent treatment for PcomA aneurysms regardless of their rupture status, with digital subtraction angiography (DSA) before treatment and high-resolution images of the ICA and PcomA to ensure reliable centerline extraction and analysis in three-dimensional (3D) reconstruction. Treatment was used as an inclusion criterion to ensure the availability of high-quality pre-treatment DSA.

Of the 184 patients initially identified, 98 patients were excluded because pre-treatment angiographic data were unavailable. During the study period, many ruptured PcomA aneurysms were treated surgically, and pre-treatment imaging was often limited to CT angiography in emergent cases. Among the remaining 86 patients with angiographic data available, 22 were further excluded because of insufficient image quality for vessel segmentation and centerline analysis, resulting in a final study cohort of 64 patients. Only one target aneurysm per patient was included in the analysis.

### 2.3. Measurement Tools

For each patient, cerebral angiography was performed before any therapeutic procedure. Using Philips workstation software (Philips, Amsterdam, The Netherlands), 3D angiographic source images were exported in digital imaging and communications in medicine (DICOM) format and reconstructed using 3D Slicer software (version 5.6.2; https://slicer.org) in stereolithography (STL) format. From the DICOM source images, vessel density was carefully adjusted, and regions affected by noise or artifacts were segmented using semi-automated methods to obtain a clean 3D vessel structure. The reconstructed 3D models were cross-validated with DSA images to ensure anatomical accuracy. The 3D Slicer platform enabled detailed manipulation and precise measurement of the reconstructed structures, including the linear and curved distances between two points, angles between intersection lines, and cross-sectional areas of selected planes in three-dimensional space.

### 2.4. Definitions of Parameters

From the reconstructed 3D structure, the morphological factors of aneurysms were measured as defined in previous studies. Commonly used parameters such as maximum size, height and neck diameter were included. We also included aspect ratio (AR; the ratio of maximum height to neck width) and size ratio (SR; the ratio of maximum height to the parent vessel diameter), as defined in previous studies [12,13]. To further evaluate aneurysm morphology, the 3D models were imported into the PyRadiomics module (version 3.1.0; https://pyradiomics.readthedocs.io/ (assessed on 13 May 2026)), and parameters such as surface area and volume were calculated in accordance with previously reported methods [14,15]. A total of 10 morphological parameters were extracted using this PyRadiomics analysis platform. All morphological factors of the aneurysms are presented in Supplementary Table S1.

In regard to morphological factors of surrounding arteries, morphological features of the ICA and PcomA from the previous studies were used. These morphological features included angulation, curvature, length and diameter [4,10,11,16]. Notably, most previous measurements of ICA or PcomA morphology have been based on 2D projections. To enhance anatomical accuracy we employed a 3D centerline-based approach to these morphologic parameters.

**Figure 1:**
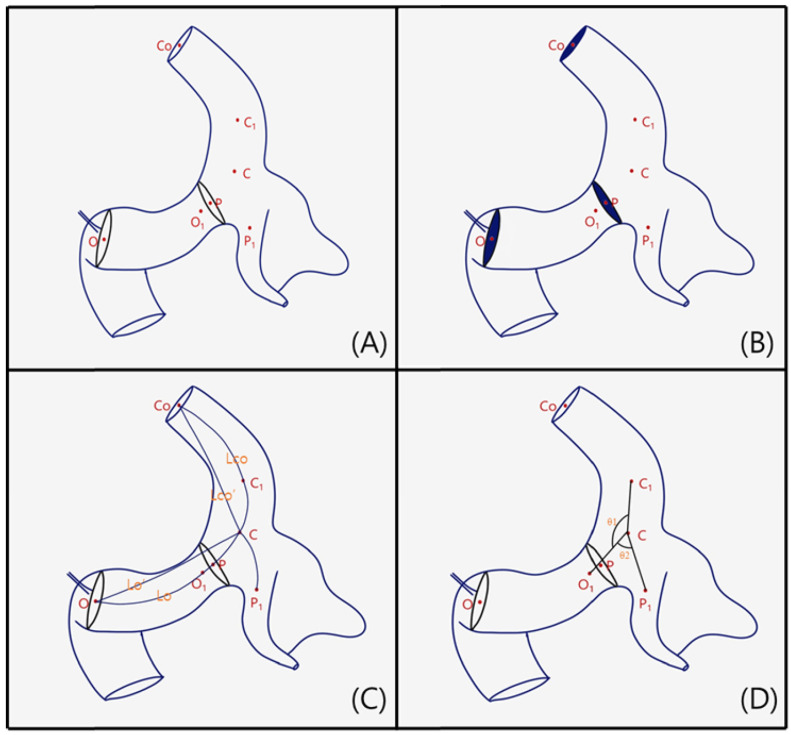
Measurement of the surrounding artery parameters. (**A**) Each plot on the central line of the internal carotid artery (ICA): O, the origin of the OphA; P, the origin of the PcomA; C, for the central point of the ICA segments and PcomA; Co, the end of the ICA; O1·P1·C1, 2 mm away from C. (**B**) Cross-section of O, P, and Co. (**C**) Lo and Lco, the center line distance; Lo′ and Lco′, the straight distance; Toc and Tcco, tortuosity of ICA ophthalmic segment and communicating segment. (**D**) θ1, angle between O1C·CC1; θ2, angle between O1C·CP1.

Along the centerline of the ICA, major branches of the ICA including PcomA and ICA bifurcation were automatically generated using the Vascular Modeling Toolkit (SlicerVMTK; version 7d22deb; http://www.apache.org/ (assessed on 13 May 2026)). The morphological parameters of the ICA and PcomA were measured based on these centerlines as Hao et al. similarly utilized centerline-derived geometry to quantify the area around the carotid siphon [3]. Measured parameters of surrounding arteries are shown in Figure 1. Key anatomical points along the centerline were defined as follows: O, representing the origin of the ophthalmic artery; P, the origin of the PcomA; C, the center point of the ICA segments and PcomA; and Co, a point just before the ICA bifurcation. Additional points were placed 2 mm along the centerline from plot C, labeled O1, P1, and C1, to assist in the angle measurements (Figure 1A). Cross-sectional areas of the vessels in plots O, P, and Co were automatically calculated through cross-sectional analyses to provide additional morphological data (Figure 1B). Curved segment lengths were defined as Lo, the distance from O to C (the ophthalmic segment of the ICA), and Lco, the distance from C to Co (the communicating segment of the ICA). Tortuosity was calculated using the equation: Tortuosity = 1 − (linear distance/distance along the branch). Curvature was defined as the inverse of the diameter of the osculating circle fitting Lo and Lco (Figure 1C). The angles were measured based on the centerline orientation, where θ1 represented the angle between segments O1C and CC1, and θ2 represented the angle between segments O1C and CP1 (Figure 1D). The definitions of the surrounding artery parameters are presented in Supplementary Table S2. Our data was collected by three observers (two neurosurgeons and one nurse researcher). Subsequent calculation of parameters was performed in a semi-automated method using VMTK, which further reduced the influence of manual variability. To assess inter-rater reliability, three observers independently placed centerlines and centerline plots on a subset of 10 cases. The intraclass correlation coefficient (ICC) was calculated. ICC values were 0.92 for Lo, 0.97 for θ2 and 0.76 for the Pcs, indicating generally robust reproducibility of the morphological parameters across observers.

### 2.5. Statistical Analysis

All statistical analyses were performed using SPSS software (version 28.0.1.1 (14), IBM, Chicago, IL, USA). For normally distributed measurement data, the values are presented as “mean ± standard deviation.” Univariate analyses (independent *t*-test; chi-square test) were performed to screen candidate variables. Because time-to-event data were not available, binary logistic regression was used with rupture status as the outcome variable to examine associations between aneurysm-related and surrounding arterial morphological parameters and PcomA aneurysm rupture. Receiver operating characteristic (ROC) curve analysis was conducted to evaluate discriminative performance.

To reduce the risk of overfitting in the multivariate model, predictor variables were selected based on their significance in univariate analysis (*p* < 0.1), and the number of included variables was limited according to the commonly accepted rule of at least 10 outcome events per predictor variable (EPV). Based on the number of rupture events in our cohort, a maximum of four variables were included in the final regression model. A *p*-value < 0.05 was considered statistically significant for all tests.

## 3. Results

### 3.1. Characteristics of the Patients with PcomA Aneurysm

A total of 184 patients with PcomA aneurysms who underwent treatment at a single tertiary medical center between January 2018 and December 2022 were reviewed. Of these patients, 64 met the inclusion criteria. The mean age of the patients was 65 ± 12 years with 55 females (86%) and 9 males (14%). More than half of the patients had underlying hypertension (HTN) (45 patients, 70%), 23 (36%) had hyperlipidemia, and only two had a family history of SAH. The mean size of the PcomA aneurysms was 6.8 ± 3.6 mm, and 19 (30%) aneurysms contained blebs. There were 25 and 39 patients with ruptured and unruptured PcomA aneurysms, respectively (Table 1). Table 2 shows the general characteristics of patients in ruptured and unruptured groups. There were no statistical differences in age (66 ± 11 vs. 63 ± 12 years, *p* = 0.185), sex (10.3% vs. 20.0%, *p* = 0.274), HTN status (74.4% vs. 64%, *p* = 0.376), family history (5.1% vs. 0%, *p* = 0.25), smoking status (17.9% vs. 16%, *p* = 0.84) and body mass index (22.91 ± 3.55 vs. 22.79 ± 3.11 kg/m^2^, *p* = 0.446) between the two groups.

### 3.2. Morphological Parameters of the Aneurysms

Seventeen factors, including the measured and radiomic parameters, were analyzed as morphological parameters of the aneurysms. These included: Bleb, Size max (Smax), Height, Neck, Height/Width (HW), AR, SR, Elongation, Flatness, Maximum 2D Diameter Column, Maximum 2D Diameter Row, Maximum 2D Diameter Slice, Maximum 3D Diameter, Mesh Volume, Sphericity, Surface Area, and Surface Volume Ratio. Table 3 presents the results of the univariate analysis comparing the morphological parameters of ruptured and unruptured aneurysms. Nine parameters showed significant differences in rupture status. Ruptured aneurysms were larger in Smax (7.919 ± 4.017 mm vs. 6.072 ± 3.059 mm, *p* = 0.021), height (6.452 ± 3.412 mm vs. 4.806 ± 2.799 mm, *p* = 0.02), and AR (1.513 ± 0.702 vs. 1.172 ± 0.493, *p* = 0.013). The Maximum 2D Diameter Column (7.503 ± 3.362 mm vs. 5.897 ± 2.448 mm, *p* = 0.016), Maximum 2D Diameter Row (8.151 ± 4.177 mm vs. 5.912 ± 2.935 mm, *p* = 0.007), and Maximum 2D Diameter Slice (7.62 ± 3.362 mm vs. 5.973 ± 2.996 mm, *p* = 0.023) were higher in the ruptured aneurysm group. The Maximum 3D Diameter (10.874 ± 6.247 mm vs. 6.636 ± 3.009 mm, *p* = 0.002) was greater in the ruptured aneurysm group. In addition, the Surface Area (156.476 ± 136.921 mm^2^ vs. 99.89 ± 100.984 mm^2^, *p* = 0.031) and Surface Volume Ratio (1.58 ± 0.722 vs. 1.972 ± 0.951, *p* = 0.042) were larger in the ruptured aneurysm group.

### 3.3. Morphological Parameters of the ICAs

Thirteen ICA parameters (Lo, CURo, Toc, Lco, CURc, Tcco, Ocs, Pcs, Cocs, θ1, θ2, Rpo, and Rcop) were evaluated. Table 4 provides the results of the univariate analysis comparing the ICA morphological parameters of ruptured and unruptured aneurysms. Tcco (0.08 ± 0.074 vs. 0.043 ± 0.034, *p* = 0.013) and Lco (7.637 ± 3.305 mm vs. 6.116 ± 1.471 mm, *p* = 0.019) were prominently large in the ruptured aneurysm group. Pcs (12.579 ± 3.886 mm^2^ vs. 10.405 ± 3.163 mm^2^, *p* = 0.011), Cocs (9.917 ± 3.18 mm^2^ vs. 8.134 ± 1.41 mm^2^, *p* = 0.002) and Rpo (0.927 ± 0.316 vs. 0.73 ± 0.188, *p* = 0.003) were larger in the unruptured aneurysm group. In addition, θ2 (87.277 ± 12.357 degrees vs. 79.452 ± 12.534 degrees, *p* = 0.008) was higher in the unruptured aneurysm group.

### 3.4. Combined Morphological Parameters of the Aneurysms and ICAs

Variables that showed statistical significance in univariate analyses were entered into the multivariate logistic regression model. Following multivariate analysis, Maximum 3D Diameter was the only aneurysm-related morphological parameter that remained significantly associated with rupture of PcomA aneurysms (b = 0.239, *p* = 0.0031). On the other hand, three parameters, namely larger Tcco (b = 15.897, *p* = 0.0344), smaller Pcs (b = −0.2179, *p* = 0.0224), and smaller θ2 (b = −0.0533, *p* = 0.0407), were identified as the significant factors based on ICA morphology associated with the rupture of PcomA aneurysms (Table 5). No significant multicollinearity was identified among the final predictors, with all variance inflation factor values below 1.1.

**Figure 2:**
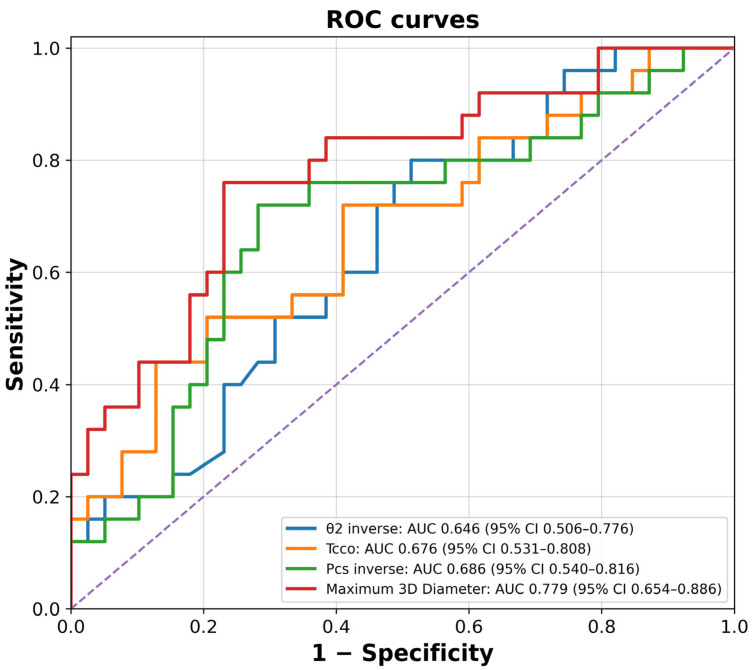
Receiver operating characteristic (ROC) curves for the established factors. A larger area under the curve (AUC) indicates greater sensitivity and specificity of that factor in distinguishing between the ruptured and unruptured groups. θ2_inv (−θ2) and Pcs_inv (1/Pcs). θ2, angle between O1C and CP1; Pcs, cross-sectional area of the cross-section of P; Tcco, tortuosity of the CCo center line.

ROC curve analysis was conducted to identify the potential of both aneurysm and ICA morphological parameters in predicting the rupture of PcomA aneurysms (Figure 2). Maximum 3D Diameter had the largest area under the curve (AUC) (0.779, 95% confidence interval (CI)), and the cut-off point was 7.805 mm. Tcco demonstrated an AUC of 0.676 (95% CI), and the cut-off point was 0.0446. Pcs and θ2 had negative relationships with the rupture status; therefore, the ROC curves were drawn with Pcs_inverse (1/Pcs) and θ2_inverse (−θ2). Pcs demonstrated an AUC of 0.686 (95% CI) with a cut-off point of 10.66 mm^2^. θ2 demonstrated an AUC of 0.646 (95% CI) with a cut-off point of 84.25 degrees.

## 4. Discussion

Current clinical practice generally follows the PHASES scoring system in deciding the treatment for unruptured cerebral aneurysms, which emphasizes the morphological factors of patients and the aneurysms [17]. The morphological factors of aneurysms, such as the shape, size, growth dynamics, and location, are key considerations for predicting rupture risk and guiding treatment decisions [12,18,19]. Previous studies on PcomA aneurysms have primarily focused on aneurysm-centered morphology and intra-aneurysmal hemodynamics in relation to rupture risk [20,21,22]. However, rupture risk may not be fully explained by aneurysm-specific factors alone. Based on this consideration, we hypothesized that morphology associated with hemodynamic characteristics of the surrounding arteries could provide additional information relevant to aneurysm rupture. Consistent with this concept, recent studies have increasingly highlighted the role of surrounding arterial morphology in aneurysm formation and rupture [3,16,23,24,25]. In particular, Hao et al. demonstrated that morphological characteristics of the ICA, including increased tortuosity of the ICA segment around PcomA, are associated with PcomA aneurysm formation [3]. Building upon this conceptual framework, the present study extends these observations by evaluating both aneurysm morphology and surrounding arterial morphological parameters in relation to aneurysm rupture risk rather than aneurysm formation. Importantly, the multivariate analyses in the present study were designed to explore potential associations and to identify candidate aneurysm and surrounding arterial morphologic parameters, rather than to establish a fully validated clinical prediction model at this stage.

The Maximum 3D Diameter was significantly larger in the ruptured aneurysm group than in the unruptured aneurysm group in our study. Other morphological factors of aneurysms showed a tendency to be larger in the ruptured aneurysm group (AR, SR, etc.) but did not demonstrate statistical significance following the multivariate analysis. This result once again shows that aneurysm size can affect rupture, and larger PcomA aneurysms are more prone to rupture. It is widely accepted that larger aneurysms are more likely to rupture than smaller aneurysms [17,18,26]. Size-related parameters have also been emphasized in PcomA aneurysm ruptures. Jiang et al. found that larger size, higher AR, SR, bottleneck factor, and bleb formation could discriminate rupture-prone PcomA aneurysms. The mean size of their ruptured PcomA aneurysms was 5.83 mm [5]. From the ROC curve, the cut-off point of the Maximum 3D Diameter in this study was 7.805 mm, which is slightly larger than that reported in previous studies regarding aneurysm rupture [6,27]. This result may stem from the difference in definition between maximum size and Maximum 3D Diameter. Maximum 3D Diameter, calculated by a radiomics program using a 3D structure, is defined as the largest pairwise Euclidean distance between any surface mesh vertices, whereas conventional maximum size is simply measured from 2D structure [14]. The larger values of the Maximum 3D Diameter compared to the maximum size of aneurysms in previous radiomics-related studies further support our findings [15,28].

**Figure 3:**
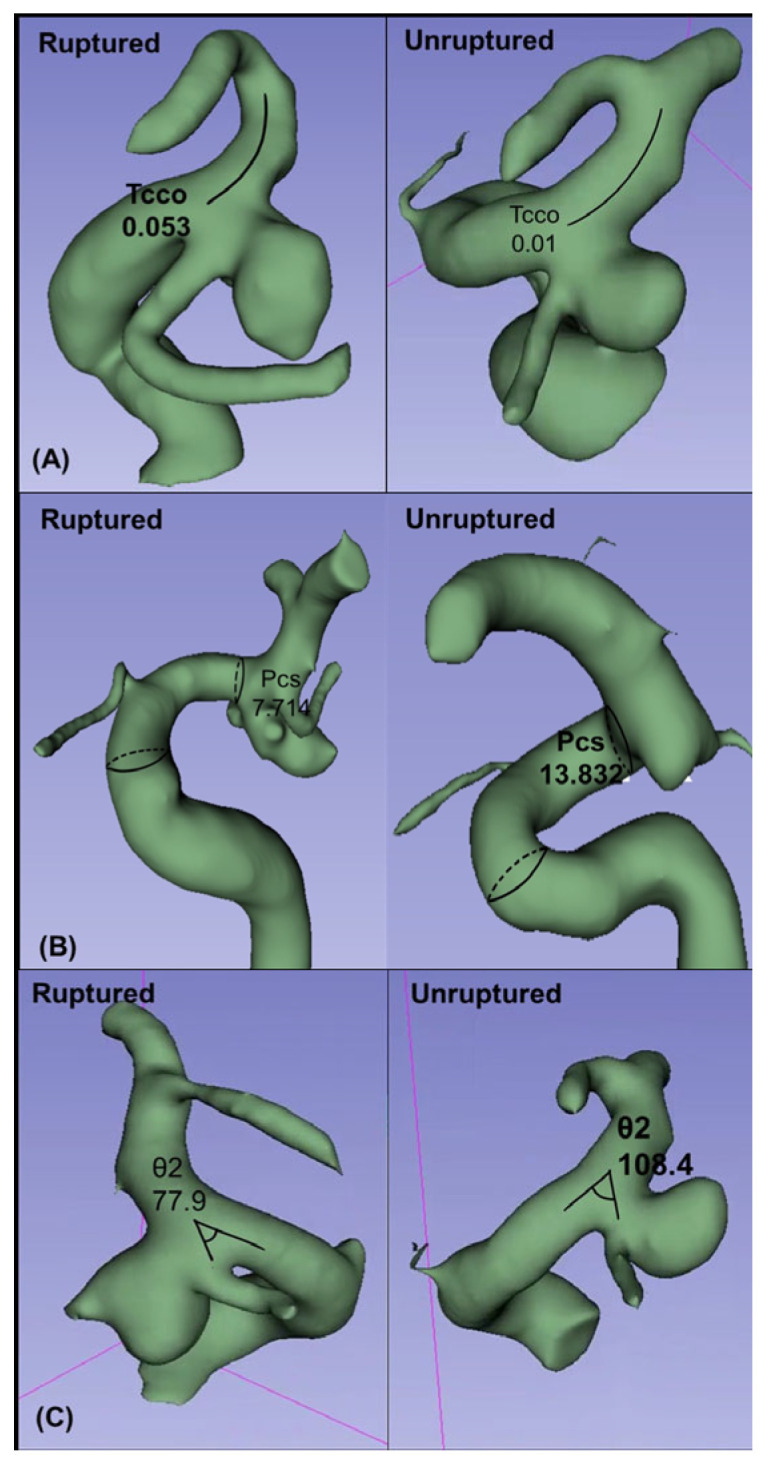
Comparison of the ICA parameters between the ruptured and unruptured aneurysm groups. (**A**) Tcco, showing that the ruptured aneurysm group has a larger tortuosity of communicating segment. (**B**) Pcs (mm^2^), showing that the ruptured aneurysm group has a smaller area in the PcomA originating site. (**C**) θ2 (°), showing that the ruptured aneurysm group has a higher angle change between the ICA and PcomA.

**Figure 4:**
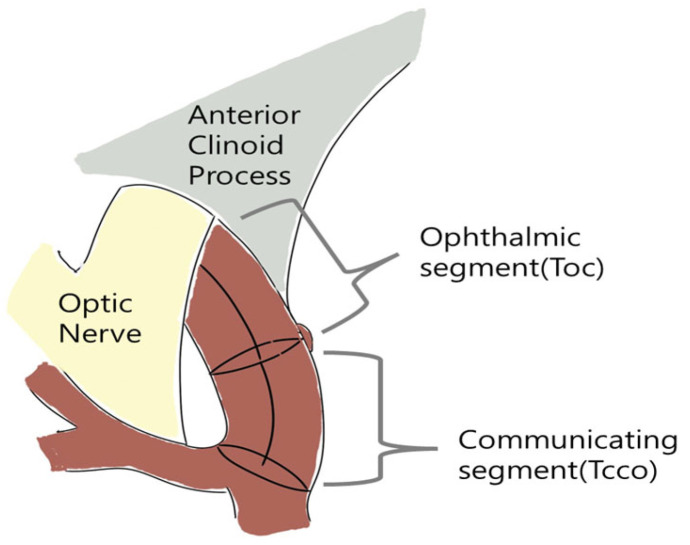
Difference between the Toc and Tcco. The ophthalmic segment is more attached to the adjacent structures.

In this study, Tcco, Pcs, and θ2 were identified as the surrounding artery parameters that may affect the rupture of PcomA aneurysms. Larger Tcco and smaller Pcs and θ2 were associated with PcomA aneurysm rupture. A larger Tcco, which represents the tortuosity of the ICA communicating segment, was associated with ruptured aneurysms. Figure 3A shows a representative case of this result. Tcco of the ruptured aneurysm (Tcco = 0.053) was greater than that of the unruptured aneurysm (Tcco = 0.01). Greater Tcco values correspond to increased curvature of the communicating segment of the ICA (comICA). Curved vessels have been shown to disrupt blood flow, causing rapid focal acceleration and turbulent flow [29,30]. Focally accelerated and turbulent flow can generate more stress in the surrounding structure. Consequently, giving more stress to surrounding structures, a more curved comICA can generate a high rupture risk of PcomA aneurysms. Similarly, Hao et al. showed that the tortuosity of the ICA segment around the PcomA contributes to the formation of PcomA aneurysms and that highly curved arteries provide more hemodynamic stress to the vessel wall, causing aneurysm initiation, growth, and rupture [3]. The tortuosity of the ophthalmic segment of the ICA (Toc) did not exhibit a significant difference regarding rupture status in our result. This may be due to the located factor of the ophthalmic segment of the ICA (ophICA). The ophICA is placed in proximity to anatomical structures, such as the anterior clinoid process and the optic nerve (Figure 4). These adjacent structures may limit the variability in the curvature of the ophICA compared with that of the comICA. The comICA, free of adjacent structures, showed greater tortuosity and variability and, as a result, showed potency as a risk factor for PcomA aneurysm rupture.

A smaller Pcs, which represents the cross-section of the ICA where the PcomA originates, was associated with ruptured aneurysms in this study. Figure 3B shows that the Pcs of the ruptured aneurysms (Pcs = 7.714) was lower than that of the unruptured aneurysms (Pcs = 13.832). Similarly, Huhtakangas et al. found that the ICA diameter at the skull base was smaller in ruptured aneurysms, which is consistent with the results of our study [4]. According to Bernoulli’s principle of fluid dynamics, the flow velocity is inversely proportional to the cross-sectional area when a fluid flows through a tube with varying cross-sectional areas. Although blood flow differs from ideal fluid behavior due to its compressibility, viscosity, and pulsatile nature, the basic principle still provides valuable insights [31]. A smaller Pcs or a reduced cross-sectional area at the origin of the PcomA may lead to an overall increase in blood flow velocity around the aneurysm. The increased velocity of the surrounding blood flow can result in higher average flow velocities entering the aneurysm. This elevated hemodynamic stress may be associated with aneurysm rupture status [32]. θ2, which represents the angle between the ICA and PcomA, was found to be smaller in the ruptured aneurysm group. Figure 3C shows that the θ2 of the ruptured aneurysms (θ2 = 77.9°) was smaller than that of the unruptured aneurysms (θ2 = 108.4°). Other research demonstrated that θ2 showed a statistical difference between the aneurysmal and normal arteries, and a lower θ2 was associated with aneurysm formation [11]. Gao et al. explained that branches forming a smaller lateral angle with the parent vessel had significantly greater hemodynamic stresses. This finding provides a reasonable explanation for our results [29].

Interpretation of these morphologic findings requires consideration of the study population. The present study included only treated PcomA aneurysms, which inevitably resulted in a clinically enriched cohort of aneurysms judged to be relevant or at elevated rupture risk. Within this clinically selected cohort, morphologic differences related to surrounding arterial anatomy could be more readily identified, allowing assessment of which parameters were associated with rupture status. Notably, despite this selection bias, aneurysm size remained strongly associated with rupture status, underscoring its central role in rupture risk assessment while also reflecting current clinical practice in which unruptured aneurysms are treated in a preventive manner with respect to size.

Previous computational fluid dynamics studies on PcomA aneurysms have primarily focused on intra-aneurysmal hemodynamics, demonstrating strong associations between rupture risk and parameters such as low wall shear stress within the aneurysm sac [20,21]. However, the role of the surrounding parent artery in shaping these hemodynamic environments has been less directly examined. The surrounding artery parameters (Tcco, Pcs, and θ2) may reflect altered hemodynamic conditions around the aneurysm rather than directly causing rupture. A series of computational fluid dynamics studies regarding intracranial vessels have demonstrated that elevated wall shear stress (WSS) and oscillatory shear index are related to hemodynamic stress [33,34,35,36,37]. As observed by Yang et al., increased WSS and strain in the parent vessel may lead to hemodynamic stress on the vessel wall, thereby promoting aneurysm formation [37]. WSS can be estimated using Poiseuille’s law (τ_s_: Frictional force per unit area on the inner vessel wall; *R*: radius of the vessel; Q: blood flow; μ: blood viscosity) [38].

$$\tau_{s}=\frac{4\mu Q}{\pi R^{3}}$$

As shown in the equation, the shear stress is strongly dependent on blood velocity, which can be affected by vascular geometry. In this context, surrounding arterial morphologic parameters may serve as surrogate markers reflecting altered hemodynamic conditions, rather than direct measures of flow or wall shear stress. However, direct assessment of flow dynamics or wall shear stress was not performed in this study, and dedicated computational hemodynamic analysis will be required to validate these mechanistic concepts. Morphological parameters of aneurysms are likely to remain the primary determinants in guiding the management of unruptured cerebral aneurysms. However, our findings suggest that surrounding arterial morphology may provide adjunctive information in selected cases, particularly when aneurysm characteristics alone appear insufficient to explain rupture risk. We presented cut off values (Tcco > 0.0446, Pcs < 10.66mm^2^ and θ2 < 84.25°) to illustrate potential thresholds associated with rupture status; however, these values should be interpreted as exploratory and require validation in larger, prospective cohorts before clinical application.

The main limitation of our study lies in the small sample size and the limited statistical power to support our findings. We recognize the restricting enrollment to patients with PcomA aneurysms and requiring high-quality imaging for parameter evaluation further reduced the sample size. Internal validation demonstrated acceptable discrimination and calibration; however, these results should be interpreted as preliminary given the limited sample size and number of rupture events. Increasing the sample size or conducting a prospective study may strengthen the reliability of the identified predictors for aneurysm rupture risk.

Another important limitation is the potential for selection bias and the restricted generalizability of our findings. First, the requirement for high-quality DSA imaging may have preferentially selected patients with more stable clinical conditions and may not fully represent the typical ruptured PcomA aneurysms. In many clinical settings, patients with poor neurologic status or emergent presentations may undergo only CTA evaluation before treatment, potentially limiting inclusion in our study cohort. Second, only patients who underwent surgical or endovascular treatment were included, introducing additional treatment-selection bias. Therefore, the identified associations should be interpreted within this clinically selected population and primarily viewed as exploratory findings regarding the potential contribution of surrounding arterial morphology to aneurysm rupture. In addition, although we applied the EPV rule when constructing the multivariate model, including four predictors with only 25 rupture events may still raise concerns of overfitting. Accordingly, the multivariate findings should be interpreted with caution and regarded as exploratory, serving primarily to highlight the potential role of ICA morphological parameters and to propose methodology for their quantitative assessment, rather than to establish a validated predictive model. Furthermore, multiple univariate comparisons were performed without formal correction for multiplicity, which may increase the risk of false positives, particularly for parameters with borderline statistical significance.

## 5. Conclusions

Our study shows that the aneurysm’s Maximum 3D Diameter, tortuosity of the communicating segment of the ICA (Tcco), cross-section of the ICA where the PcomA originates (Pcs), and the angle between the ophthalmic segment of the ICA and PcomA (θ2) were associated with rupture status of PcomA aneurysms. Furthermore, the morphological factors of the surrounding artery may provide complementary information to aneurysm-based assessment and could assist clinical decision-making in selected cases. These findings suggest that surrounding arterial morphology may contribute additional information for individualized rupture-risk assessment beyond conventional aneurysm-centered evaluation. Further prospective studies with larger cohorts are warranted to validate the clinical applicability of these morphological parameters.

## Figures and Tables

**Table 1:** Baseline patient and aneurysm characteristics.

Variable	Frequency
Patient demographics, *n* = 64	
Age, mean ± standard deviation	65 ± 12
Female, *n* (%)	55 (86)
Medical history, *n* (%)	
Hypertension	45 (70)
Hyperlipidemia	23 (36)
Family history of SAH	2 (3.1)
Smoking	11 (17.2)
Aneurysm characteristics, *n* = 64	
Size, mean ± standard deviation, mm	6.8 ± 3.6
Ruptured status, *n* (%)	25 (39)
Bleb, *n* (%)	19 (30)

**Table 2:** Univariate analysis of the general characteristics.

Parameter	Unruptured (39)	Ruptured (25)	*p*-Value
Age	66.103 ± 10.765	63.48 ± 12.128	0.185
Sex (Male)	4 (10.3%)	5 (20.0%)	0.274
HTN	29 (74.4%)	16 (64.0%)	0.376
Family history	2 (5.1%)	0 (0.0%)	0.250
Hyperlipidemia	15 (38.5%)	8 (32.0%)	0.599
Smoking	7 (17.9%)	4 (16.0%)	0.840
BMI	22.911 ± 3.55	22.792 ± 3.116	0.446

Abbreviations: HTN, hypertension; BMI, body mass index.

**Table 3:** Univariate analysis of the morphological parameters of the aneurysms.

Parameter	Unruptured (39)	Ruptured (25)	*p*-Value
Bleb	12 (30.8%)	7 (28.0%)	0.813
Smax	6.072 ± 3.059	7.919 ± 4.017	0.021 *
Height	4.806 ± 2.799	6.452 ± 3.412	0.020 *
Neck	3.96 ± 1.412	4.339 ± 1.68	0.167
HW	1.113 ± 0.265	1.133 ± 0.238	0.383
AR	1.172 ± 0.493	1.513 ± 0.702	0.013 *
SR	1.603 ± 3.05	2.65 ± 5.689	0.171
Elongation	0.761 ± 0.114	0.712 ± 0.161	0.080
Flatness	0.617 ± 0.103	0.573 ± 0.176	0.227
M-2D Diameter Column	5.897 ± 2.448	7.503 ± 3.384	0.016 *
M-2D Diameter Row	5.912 ± 2.935	8.151 ± 4.177	0.007 *
M-2D Diameter Slice	5.973 ± 2.996	7.62 ± 3.362	0.023 *
M-3D Diameter	6.636 ± 3.009	10.874 ± 6.247	0.002 *
Mesh Volume	91.566 ± 133.66	163.195 ± 207.86	0.067
Sphericity	0.807 ± 0.046	0.793 ± 0.04	0.108
Surface Area	99.89 ± 100.984	156.476 ± 136.921	0.031 *
Surface Volume Ratio	1.972 ± 0.951	1.58 ± 0.722	0.042 *

Abbreviations: AR, aspect ratio; SR, size ratio; M-2D, maximum 2D; M-3D, maximum 3D; HW, ratio of height and width. * *p* < 0.05.

**Table 4:** Univariate analysis of the morphological parameters of the internal carotid artery.

Parameter	Unruptured (39)	Ruptured (25)	*p*-Value
Lo	9.881 ± 2.655	10.094 ± 2.021	0.367
CURo	0.214 ± 0.058	0.235 ± 0.05	0.073
Toc	0.103 ± 0.077	0.117 ± 0.052	0.222
Lco	6.116 ± 1.471	7.637 ± 3.305	0.019 *
CURc	0.178 ± 0.079	0.19 ± 0.068	0.279
Tcco	0.043 ± 0.034	0.08 ± 0.074	0.013 *
Ocs	14.057 ± 3.519	14.607 ± 3.797	0.278
Pcs	12.579 ± 3.886	10.405 ± 3.163	0.011 *
Cocs	9.917 ± 3.185	8.134 ± 1.41	0.002 *
θ1	44.538 ± 12.778	46.368 ± 13.68	0.294
θ2	87.277 ± 12.357	79.452 ± 12.534	0.008 *
Rpo	0.927 ± 0.316	0.73 ± 0.188	0.003 *
Rcop	0.823 ± 0.263	0.841 ± 0.257	0.394

Abbreviations: Lo, centerline distance from O to C; CURo, diameter of the osculating circle fitting Lo; Toc, tortuosity of the OC center line; Lco, centerline distance from C to Co; CURc, diameter of the osculating circle fitting Lco; Tcco, tortuosity of the CCo center line; Ocs, cross-sectional area of the cross-section of O; Pcs, cross-sectional area of the cross-section of P; Cocs, cross-sectional area of the cross-section of Co; θ1, angle between O1C and CC1; θ2, angle between O1C and CP1; Rpo, ratio between Ocs and Pcs (Pcs/Ocs); Rcop, ratio between Pcs and Cocs (Cocs/Pcs). * *p* < 0.05.

**Table 5:** Multivariate analysis of the parameters of the aneurysms and internal carotid artery.

Parameter	B	*p*-Value
Maximum 3D Diameter	0.23868	0.0031
Tcco	15.8969	0.0344
Pcs	−0.21794	0.0224
θ2	−0.05331	0.0407

## Data Availability

The PyRadiomics scripts used for feature extraction are available upon request. Due to patient confidentiality and institutional regulations, imaging data cannot be J. Clin. Med. 2025, 14, 3682 11 of 12 shared publicly but may be made available in de-identified form upon reasonable request and with appropriate ethical approval.

## Supplementary Materials

The following supporting information can be downloaded at: https://www.mdpi.com/article/10.3390/jcm15103783/s1, Table S1: Morphological parameters of the aneurysm, Table S2: Morphological parameters of the surrounding artery.

## Abbreviations

The following abbreviations are used in this manuscript:

SAH	Subarachnoid hemorrhage
PcomA	Posterior communicating artery
AR	Aspect ratio
SR	Size ratio
ICA	Internal carotid artery
3D	Three dimensional
2D	Two dimensional
DSA	Digital subtraction angiography
ROC	Receiver operating characteristic
HTN	Hypertension
DM	Diabetes mellitus
AUC	Area under the curve

## References

[1] Van Gijn J., Kerr R.S., Rinkel G.J. (2007). Subarachnoid haemorrhage. Lancet.

[2] National Clinical Research Center for Neurological, Society of Neurosurgery of Chinese Medical Association, Society of Cerebrovascular Surgery of Chinese Stroke Association (2024). Chinese guideline for the clinical management of patients with unruptured intracranial aneurysms. Zhonghua Yi Xue Za Zhi.

[3] Hao W., Hao H., Ren C.F., Wang X., Gao B. (2022). Associations Between Posterior Communicating Artery Aneurysms and Morphological Characteristics of Surrounding Arteries. Front. Neurol..

[4] Huhtakangas J., Lehecka M., Lehto H., Jahromi B.R., Niemela M., Kivisaari R. (2017). CTA analysis and assessment of morphological factors related to rupture in 413 posterior communicating artery aneurysms. Acta Neurochir..

[5] Jiang H., Shen J., Weng Y.X., Pan J.W., Yu J.B., Wan Z.A., Zhan R. (2015). Morphology Parameters for Mirror Posterior Communicating Artery Aneurysm Rupture Risk Assessment. Neurol. Med. Chir..

[6] Lv N., Feng Z., Wang C., Cao W., Fang Y., Karmonik C., Liu J., Huang Q. (2016). Morphological Risk Factors for Rupture of Small (<7mm) Posterior Communicating Artery Aneurysms. World Neurosurg..

[7] Skodvin T.O., Johnsen L.H., Gjertsen O., Isaksen J.G., Sorteberg A. (2017). Cerebral Aneurysm Morphology Before and After Rupture: Nationwide Case Series of 29 Aneurysms. Stroke.

[8] Yi J., Zielinski D., Chen M. (2016). Cerebral Aneurysm Size before and after Rupture: Case Series and Literature Review. J. Stroke Cerebrovasc. Dis..

[9] Meng H., Tutino V.M., Xiang J., Siddiqui A. (2014). High WSS or Low WSS? Complex Interactions of Hemodynamics with Intracranial Aneurysm Initiation, Growth, and Rupture: Toward a Unifying Hypothesis. AJNR Am. J. Neuroradiol..

[10] Rosato R., Comptdaer G., Mulligan R., Breton J.M., Lesha E., Lauric A., Malek A.M. (2020). Increased focal internal carotid artery angulation in patients with posterior communicating artery aneurysms. J. NeuroInterv. Surg..

[11] Yu M., Huang Q., Hong B., Qiao F., Liu J. (2010). Morphological differences between the aneurysmal and normal artery in patients with internal carotid-posterior communicating artery aneurysm. J. Clin. Neurosci..

[12] Dhar S., Tremmel M., Mocco J., Kim M., Yamamoto J., Siddiqui A.H., Hopkins L.N., Meng H. (2008). Morphology parameters for intracranial aneurysm rupture risk assessment. Neurosurgery.

[13] Raghavan M.L., Ma B., Harbaugh R.E. (2005). Quantified aneurysm shape and rupture risk. J. Neurosurg..

[14] Liu Q., Jiang P., Jiang Y., Ge H., Li S., Jin H., Li Y. (2019). Prediction of Aneurysm Stability Using a Machine Learning Model Based on PyRadiomics-Derived Morphological Features. Stroke.

[15] Ludwig C.G., Lauric A., Malek J.A., Mulligan R., Malek A.M. (2021). Performance of Radiomics derived morphological features for prediction of aneurysm rupture status. J. NeuroInterv. Surg..

[16] Labeyrie P.E., Gory B., Huguet N., Grenier C., Ditac G., Sadeh-Gonik U., Riva R., Turjman F. (2016). Carotid siphon morphology: Is it associated with posterior communicating aneurysms?. Interv. Neuroradiol..

[17] Greving J.P., Wermer M.J., Brown R.D., Morita A., Juvela S., Yonekura M., Ishibashi T., Torner J.C., Nakayama T., Rinkel G.J. (2014). Development of the PHASES score for prediction of risk of rupture of intracranial aneurysms: A pooled analysis of six prospective cohort studies. Lancet Neurol..

[18] Lee U.Y., Kwak H.S. (2021). Analysis of Morphological-Hemodynamic Risk Factors for Aneurysm Rupture Including a Newly Introduced Total Volume Ratio. J. Pers. Med..

[19] Li M., Hu S., Yu N., Zhang Y., Luo M. (2019). Association Between Meteorological Factors and the Rupture of Intracranial Aneurysms. J. Am. Heart Assoc..

[20] Castiglione J.A., Drake A.W., Hussein A.E., Johnson M.D., Palmisciano P., Smith M.S., Robinson M.W., Stahl T.L., Jandarov R.A., Grossman A.W. (2023). Complex Morphologic Analysis of Cerebral Aneurysms Through the Novel Use of Fractal Dimension as a Predictor of Rupture Status: A Proof of Concept Study. World Neurosurg..

[21] Duan G., Lv N., Yin J., Xu J., Hong B., Xu Y., Liu J., Huang Q. (2016). Morphological and hemodynamic analysis of posterior communicating artery aneurysms prone to rupture: A matched case-control study. J. NeuroInterv. Surg..

[22] Yuan J., Huang C., Li Z., Jiang X., Zhao X., Wu D., Lai N., Liu J., Zhang B., Qin F. (2021). Hemodynamic and Morphological Parameters of Ruptured Mirror Posterior Communicating Artery Aneurysms. Front. Neurol..

[23] Tsutsui T., Ikedo T., Kitazawa Y., Otsuka R., Nishiwaki T., Kushi Y., Niwa A., Ozaki S., Hattori E.Y., Shimonaga K. (2023). Impact of Morphological Factors on the Future Growth of Unruptured Posterior Communicating Artery Aneurysms. World Neurosurg..

[24] Xu Z., Kim B.S., Lee K.S., Choi J.H., Shin Y.S. (2019). Morphological and clinical risk factors for the rupture of posterior communicating artery aneurysms: Significance of fetal-type posterior cerebral artery. Neurol. Sci..

[25] Han K., Nahm M., Ko S.W., Yi H.J., Chun H.J., Lee Y.J., Lee S.H., Ryu J., Song S., Choi K.S. (2025). Influence of Fetal-Type Posterior Cerebral Artery on Morphological Characteristics and Rupture Risk of Posterior Communicating Artery Aneurysms: A Radiomics Approach. J. Clin. Med..

[26] Zheng Y., Xu F., Ren J., Xu Q., Liu Y., Tian Y., Leng B. (2016). Assessment of intracranial aneurysm rupture based on morphology parameters and anatomical locations. J. NeuroInterv. Surg..

[27] Xu J., Yu Y., Wu X., Wu Y., Jiang C., Wang S., Huang Q., Liu J. (2013). Morphological and hemodynamic analysis of mirror posterior communicating artery aneurysms. PLoS ONE.

[28] Turhon M., Li M., Kang H., Huang J., Zhang F., Zhang Y., Zhang Y., Maimaiti A., Gheyret D., Axier A. (2023). Development and validation of a deep learning model for prediction of intracranial aneurysm rupture risk based on multi-omics factor. Eur. Radiol..

[29] Gao B.L., Hao H., Hao W., Ren C.F., Yang L., Han Y. (2022). Cerebral aneurysms at major arterial bifurcations are associated with the arterial branch forming a smaller angle with the parent artery. Sci. Rep..

[30] Gao B.L., Hao W.L., Ren C.F., Li C.H., Wang J.W., Liu J.F. (2022). Greater hemodynamic stresses initiated the anterior communicating artery aneurysm on the vascular bifurcation apex. J. Clin. Neurosci..

[31] Hussain-Amin A., Parekh A., Mohan J. (2024). Basic Concepts of Echocardiography Hemodynamics. StatPearls.

[32] Cebral J.R., Mut F., Weir J., Putman C.M. (2011). Association of hemodynamic characteristics and cerebral aneurysm rupture. AJNR Am. J. Neuroradiol..

[33] Fattahi M., Abdollahi S.A., Alibak A.H., Hosseini S., Dang P. (2023). Usage of computational method for hemodynamic analysis of intracranial aneurysm rupture risk in different geometrical aspects. Sci. Rep..

[34] Munarriz P.M., Gómez P.A., Paredes I., Castaño-Leon A.M., Cepeda S., Lagares A. (2016). Basic Principles of Hemodynamics and Cerebral Aneurysms. World Neurosurg..

[35] Murayama Y., Fujimura S., Suzuki T., Takao H. (2019). Computational fluid dynamics as a risk assessment tool for aneurysm rupture. Neurosurg. Focus..

[36] Wong G.K., Poon W.S. (2011). Current status of computational fluid dynamics for cerebral aneurysms: The clinician’s perspective. J. Clin. Neurosci..

[37] Yang H., Kim J.-J., Kim Y.B., Cho K.-C., Oh J.H. (2024). Investigation of Paraclinoid Aneurysm Formation by Comparing the Combined Influence of Hemodynamic Parameters Between Aneurysmal and Non-Aneurysmal Arteries. J. Cereb. Blood Flow Metab..

[38] Malek A.M., Alper S.L., Izumo S. (1999). Hemodynamic shear stress and its role in atherosclerosis. JAMA.

