# Supplementary material for: Prediction of Posterior Communicating Artery Aneurysm Rupture Risk: A Multivariate Analysis of Aneurysm and Surrounding Arterial Morphological Factors
Source: J Clin Med. 2026 May 14;15(10):3783. doi: 10.3390/jcm15103783 (PMC13206929; doi:10.3390/jcm15103783)
Supplement: Supplementary file 1 [file jcm-15-03783-s001.zip › jcm-4294820-supplementary.pdf]

Supplemental Digital Content

Table S1. Morphological parameters of the aneurysm

Parameter	Definition
Maximum size	Longest distance from the aneurysm's neck plane to the dome
Height	Greatest distance from the center of the aneurysm's neck to the surface
Neck	Longest measurement of the neck plane of the aneurysm
Height/Width	Ratio of the maximum height to the width (maximum width parallel to the neck plane)
AR	Ratio of the maximum height to the neck
SR	Ratio of the maximum height to the parent vessel size
Elongation	A measure that shows the relationship between the two largest principal components in the shape
Flatness	A measure that shows the relationship between the largest and smallest principal components of the shape
M-2D Diameter Column	The largest pairwise Euclidean distance between the surface mesh vertices in the coronal plane
M-2D Diameter Row	The largest pairwise Euclidean distance between the surface mesh vertices in the sagittal plane
M-2D Diameter Slice	The largest pairwise Euclidean distance between the surface mesh vertices in the axial plane
M-3D Diameter	The largest pairwise Euclidean distance between the surface mesh vertices
Mesh Volume	The total volume of the shape
Sphericity	A measure of the roundness of the shape relative to a sphere
Surface Area	The total area of the shape
Surface Volume Ratio	The ratio of the surface area to the volume of the shape

Abbreviations: AR, aspect ratio; SR, size ratio; M-2D, maximum 2D; M-3D, maximum 3D.

Table S2. Morphological parameters of the surrounding artery

Parameter	Definition
Lo	Longest distance from the aneurysm's neck plane to the dome
CURo	Greatest distance from the center of the aneurysm's neck to the surface
Toc	Longest measurement of the neck plane of the aneurysm
Lco	Ratio of the maximum height to the width (maximum width parallel to the neck plane)
CURc	Ratio of the maximum height to the neck
Tcco	Ratio of the maximum height to the parent vessel size
Ocs	A measure that shows the relationship between the two largest principal components in the shape
Pcs	A measure that shows the relationship between the largest and smallest principal components of the shape
Cocs	The largest pairwise Euclidean distance between the surface mesh vertices in the coronal plane
θ1	The largest pairwise Euclidean distance between the surface mesh vertices in the sagittal plane
θ2	The largest pairwise Euclidean distance between the surface mesh vertices in the axial plane
Rpo	The largest pairwise Euclidean distance between the surface mesh vertices
Rcop	The total volume of the shape
